# Anterior Cruciate Ligament Tear Detection Based on Deep Convolutional Neural Network

**DOI:** 10.3390/diagnostics12102314

**Published:** 2022-09-26

**Authors:** Kavita Joshi, K. Suganthi

**Affiliations:** Vellore Institute of Technology, Chennai 600127, India

**Keywords:** anterior cruciate ligament, deep learning, deep convolutional neural network, medical image processing, tear detection

## Abstract

Anterior cruciate ligament (ACL) tear is very common in football players, volleyball players, sprinters, runners, etc. It occurs frequently due to extra stretching and sudden movement and causes extreme pain to the patient. Various computer vision-based techniques have been employed for ACL tear detection, but the performance of most of these systems is challenging because of the complex structure of knee ligaments. This paper presents a three-layered compact parallel deep convolutional neural network (CPDCNN) to enhance the feature distinctiveness of the knee MRI images for anterior cruciate ligament (ACL) tear detection in knee MRI images. The performance of the proposed approach is evaluated for the MRNet knee images dataset using accuracy, recall, precision, and the F1 score. The proposed CPDCNN offers an overall accuracy of 96.60%, a recall rate of 0.9668, a precision of 0.9654, and an F1 score of 0.9582, which shows superiority over the existing state-of-the-art methods for knee tear detection.

## 1. Introduction

Deep learning plays a crucial role in distinct medical image processing applications such as image reconstruction, lesion and tissue segmentation, and the characterization and disease detection of medical abnormality [1,2]. Deep learning is important in disease detection in clinical radiology, as it diminishes the bias and errors that occur due to fatigue and distraction. In recent years, deep learning is chiefly used for the detection of breast mammogram masses [3]; opacities, cardiomegaly, and lung nodules in chest radiographs; and interstitial lung disease and lung nodules in chest CT. However, the application of deep learning is rarely addressed for MRI images because of the complexity of the analysis and the processing of multiple sections of MRI images [4,5]. 

The anterior cruciate ligaments (ACLs) of the knee connect and stabilize the femur to the tibia bone of the leg. The knee consists of four ligaments: two internal ligaments known as anterior cruciate ligament (ACL) and posterior cruciate ligament (PCL) and two outside ligaments known as the medial collateral ligament and lateral collateral ligament. ACL tear injury is most common in sports persons and occurs due to sudden jerks, accidents, extra stretching, running, etc. [6,7]. The symptoms of ACL tear injury include swelling, pain, knee deformation, and difficulty in walking [3,4]. A radiologist’s job is to use radiological images to discover injuries such as torn ACLs. Manually interpreting knee ACL injuries, meniscus tears, and knee cartilage abnormalities from radiological pictures is a tedious task [8,9]. Different tears may cause knee osteoarthritis, osteoporosis, or knee joint replacement. Physical tests and biomarkers, X-rays, computed tomography (CT), mammography, ultrasound imaging, and magnetic resonance imaging (MRI) are all used to identify an ACL tear in the knee. As the ACL is not visible on a basic X-ray, MRI is the best option for identifying ACL tears [10]. Sprains and partial tears of the ACL can be distinguished from the complete and partial tears of the meniscus via MRI. An ACL is often a narrow band of low signal intensity that runs from the femoral end to the apex and can be visible in a single slice or numerous slices depending on the rotation of the scanning. ACL tears must be read in the coronal, sagittal, and axial planes to obtain the full picture of the ACL tear [11]. ACL injuries can be detected by analyzing the structure and orientation of the ligaments in an MRI image. ACL tears show changes in contour, and discontinuity and signal irregularity within the injured ligament [12]. Deep learning algorithms for image analytics have been widely applied in the medical imaging arena in recent years to handle problems such as segmentation, detection, classification, and diagnosis without the intervention of a radiologist. In the field of categorization and representation learning, the use of CNNs has received considerable attention in recent years. CNNs are effective classifiers that have excellent accuracies in a wide range of applications and have a large number of free parameters. However, many deep-learning frameworks provide a poor feature representation of MRI images because of the improper selection of network hyperparameters. 

This paper presents an ACL tear detection method based on a compact parallel deep convolutional neural network (CPDCNN) using knee MRI images. The significant contributions of the paper are summarized as follows:

- The investigation of CPDCNN for improving the deep feature discrimination to represent the complex features of knee MRI images for ACL tear detection;
- The performance estimation of the proposed CPDCNN using the public MRNet dataset based on various evaluation metrics such as accuracy, precision, recall rate, and the F1 score.

The rest of the paper is structured as follows: Section 2 includes prior work on ACL tear detection of knee. Section 3 provides brief information on the proposed CPDCNN for ACL tear detection. Section 4 gives a detailed information about experimental results and parameter configurations. Section 5 depicts the discussions and findings from the result. Finally, Section 6 concludes of the proposed work and provides the future scope of the proposed method to improve its performance.

## 2. Related Work

Bien et al. [13] investigated DCNNs for meniscal and ACL tear detection, which yielded an overall accuracy of 95%. Later, Lai et al. [14] utilized a combination of high-level features obtained using DCNNs and traditional handcrafted features to tackle the dilemma of high-resolution and insufficient datasets. It resulted in 90.2% and 90.15% accuracy values for the ISIC2017 and HIS2828 datasets, respectively. Subsequently, Liu et al. [15] proposed two deep CNN layers to segment the ACL from the T2 weighted MRI knee images for the detection of structural abnormalities. The area under the ROC curve for the ACL tear detection of the system was 0.98. Consequently, recurrent CNNs (R-CNNs) [16] have been used for the segmentation and detection of the meniscus region. The performance of the system depends on the segmentation of ACLs using a morphological filter, and over-segmentation or under-segmentation may lead to poor performance. Recently, deep learning models based on CNNs have been successfully presented for knee injury detection [17,18]. Very few works have been presented for ACL tear detection using a larger MRI database. Recently, various existing pre-trained deep learning architectures have been presented for knee ligament tear detection. Azcona et al. [19] investigated ResNet18 for ACL tear detection, which provided an overall accuracy of 93.40%. ResNet18 needs more than 11 M trainable parameters. Further, Kara et al. [20] presented ResNet50 for ACL tear detection in sagittal MRI images, resulting in an accuracy of 81.27%. It provided a better spatial representation of the MRI images, but the complex model resulted in 39,636,608 trainable parameters, which limits the performance of a standalone system, and therefore, the model needs more time for training as well as testing. Irmakci et al. [21,22,23] provided a comparison of various pre-trained deep learning frameworks such as ResNet, AlexNet, and GoogleNet for ACL tear detection. These pre-trained models were comparable for ACL tear detection using MRI images and resulted in an area under the curve (AUC) of 0.956 (ResNet), 0.938 (AlexNet), and 0.890 (GoogleNet). Awan et al. [24] presented ResNet14 along with the collaboration of real-time data augmentation and class balancing to tackle the problem of over-fitting in ACL tear detection. It gave 92% accuracy for five-fold cross-validation, along with 179,075 trainable parameters on a dataset having 917 MRI images. However, it suffered from an extensive training burden, and results were not presented for imbalanced classes. Tran et al. [25] presented CNNs based deep learning model to improve the generalization capability of tear detection models, which resulted in 87.5% and 87.00% accuracy values for the MRNet and KneeMRI datasets, respectively. Various deep learning frameworks have been investigated for knee tear detection using MRI images, and they have shown significant improvement over machine-learning-based knee tear detection. However, the existing architectures used for knee tear detection have complex network architectures, thus leading to larger training parameters [26,27]. The complex deeper deep learning architectures require extensive hyper-parameter tuning and need larger trainable parameters. The selection of particular convolutional filter window sizes and layers that can provide a better spatial representation of the fine and coarse texture of the knee MRI is a critical problem [28,29]. 

## 3. Proposed Methodology

Most of the traditional sequential DCNN networks consider the same filter kernel size for convolutional operation in all the layers connected one after another. However, the selection of a specific filter kernel is challenging because smaller filter windows (e.g., 3 × 3 pixels) provide a better representation of finer textures but may neglect larger texture variations in an MRI image, whereas larger filter windows (e.g., 7 × 7 pixels) provide a superior representation of coarse textures but may neglect finer texture variations.

**Figure 1:**
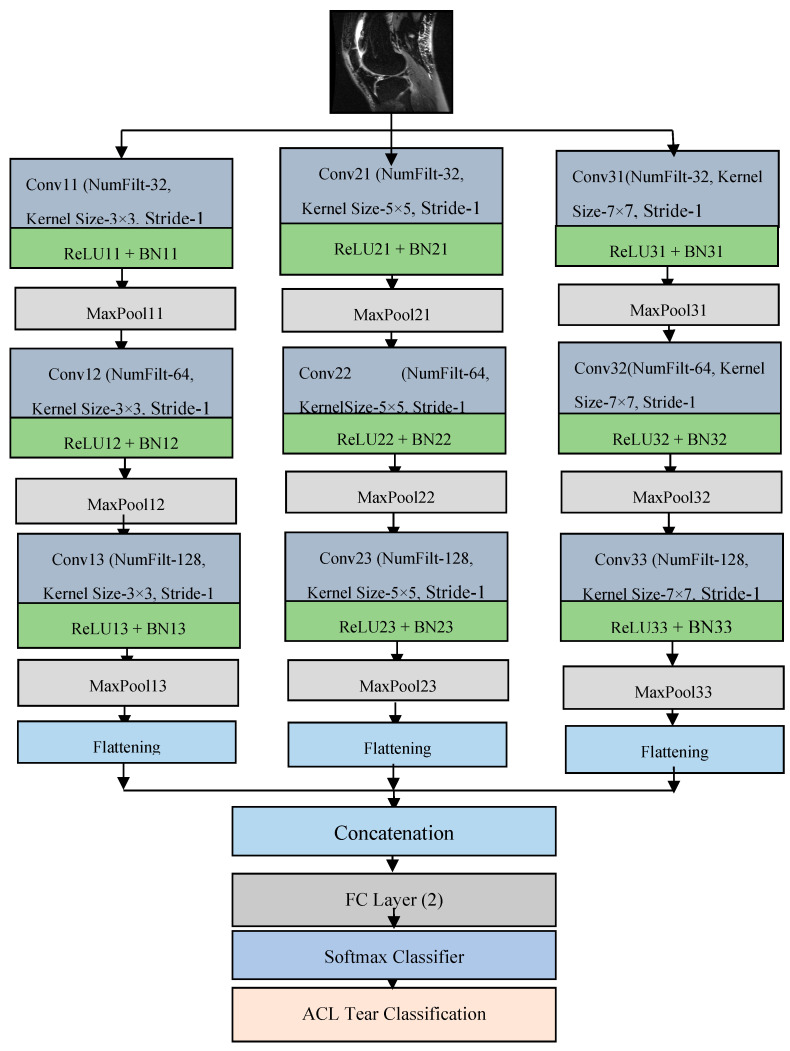
Flow diagram of proposed DCNN-based ACL tear detection.

**Figure 2:**
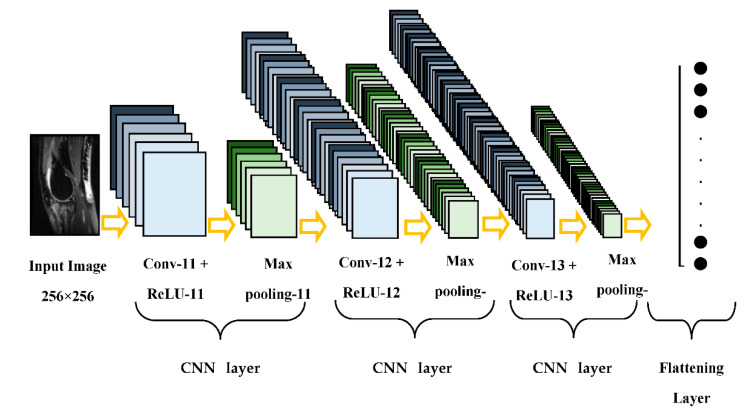
Representation of the first parallel layer of the proposed scheme.

This paper presents a compact parallel deep convolutional neural network (CPDCNN) that consists of three parallel segments of a DCNN with differentially sized filter kernels, as shown in Figure 1. The first parallel layer includes a three-layered DCNN architecture with 3 × 3 pixels convolutional filter kernels at each convolutional layer. The second and third parallel layers consist of 5 × 5 and 7 × 7 pixels convolutional filter kernels, respectively, at each convolutional layer. The proposed CPDCNN is aimed to provide a better fine and coarse texture representation of knee MRI images and minimize the computational complexity of the network. The CNN layer in each parallel segment consists of a convolutional layer (CL), a rectified linear unit layer (ReLU), a maximum pooling layer (MP), a fully connected layer, and a classification layer. Each parallel segment consists of a total of 32, 64, and 128 convolutional filters at the first, second, and third CNN layers, respectively. Figure 2 illustrates the representation of the first CNN layer of the proposed CPDCNN. 

### 3.1. Convolutional Layer

In the convolutional layer, a leg knee MRI image is convolved with convolutional filters. A convolutional layer gives a better spatial and temporal relationship between the local regions of the leg knee image signal. It provides local connectivity between the various pixels that help to learn the discriminative attributes of changes in the frequency and amplitude of the leg knee image signal indicating a tear injury. In the convolutional layer, the original knee MRI is convolved with the N number of convolutional filters. During the convolutional operation, the filter window of w × w size is multiplied with the w × w size local region of the image using element multiplication.

To capture the local connections of the leg knee image signal texture, the convolutional filter window can be moved over the complete knee MRI image. To prevent the window’s edges from disappearing, one pixel is retained in the window. Throughout the training phase, filter weights are continually updated and initialized at random. The original spectrogram is zero-padded with one pixel all around the edges to keep the image’s original size. N convolutional feature maps are produced through this convolutional procedure. Different textural characteristics of the cloth material may be learned by each filter. The intrinsic textural characteristics of the fabric material are represented by convolutional layers. Over the Whole image, the convolutional kernel advances one pixel at a time. Six convolutional kernels with a size of m × n are chosen for implementation. The initialization of each filter kernel is random. Each value on the map is called a neuron. Equations (1) and (2) provide the convolutional process for leg and knee images $I_{conv}$ using filter *F*. The original image I(m,n) having dimensions of R×C is convolved using the filter kernel F with the stride of one pixel over the rows and columns of the knee MRI image.

(1) $$I_{conv}\left(m,n\right)=I\left(m,n\right)*F$$

(2) $$I_{conv}\left(m,n\right)=\overset{R}{\underset{i=1}{\sum }}\overset{C}{\underset{j=1}{\sum }}I\left(i,j\right)F\left(i-m,j-n\right)$$

### 3.2. ReLU Layer 

The convolutional feature map’s nonlinear qualities are enhanced by the rectified linear unit. The convolutional layer’s negative values might cause the features’ nonlinear characteristics to deteriorate. The size of the feature mappings in this layer is identical to those in the convolutional layer. The negative values in convolutional feature maps are rounded to 0 in this layer, and all non-negative values are left alone to create the *ReLU* feature map ($I_{ReLU})$ as given in Equation (3).

(3) $$I_{ReLU}\left(i,j\right)=\underset{i=1:R,\;j=1:C}{\max}\{0,I_{\mathrm{conv}}\left(i,j\right)\}$$

### 3.3. Batch Normalization

The BN layer normalizes the output of the convolutional layer to reduce the internal co-variance change in the network and minimize the inter-dependency between the various layers.

### 3.4. Maximum Pooling Layer

By ignoring the less important features, the pooling layer assists in gathering the key characteristics from the *ReLU* layer feature maps. The maximum of the local window is chosen as the salient characteristic in maximum pooling. Additionally, pooling aids in reducing feature dimensions. Feature maps are scaled by a factor of (1/M) for the largest pooling window, which has M × M dimensions. For the nonoverlapping window of size M × M, pooling is performed. Equation (4) may be used to calculate the maximum pooling (*I_MP_*) for the *ReLU* layer output with a dimension of R × C.

(4) $$I_{MP}\left(i,j\right)=\underset{i=1:R-1,\;j=1:C-1}{\max}I_{ReLU}\left(i:i+1,j:j+1\right)$$

### 3.5. Fully Connected Layer

In a fully connected layer, each neuron of one layer is connected to all other neurons of the other layers to give a deeper representation of the leg knee image. 

### 3.6. Softmax Layer

To determine the class of an unknown sample, a classification layer called Softmax is used. The probabilities for each class are provided, and the class label with the highest probability is chosen as the output class. Since the Adam optimization technique requires less memory and has simple computing requirements, it is utilized to learn the CPDCNN algorithm. It has a learning rate of 0.001, decay rates of 0.9 and 0.999, and a small positive parameter $\varepsilon =10^{-8}$ to avoid division by zero [19].

## 4. Experimental Results

### 4.1. System Configurations and Dataset

**Figure 3:**
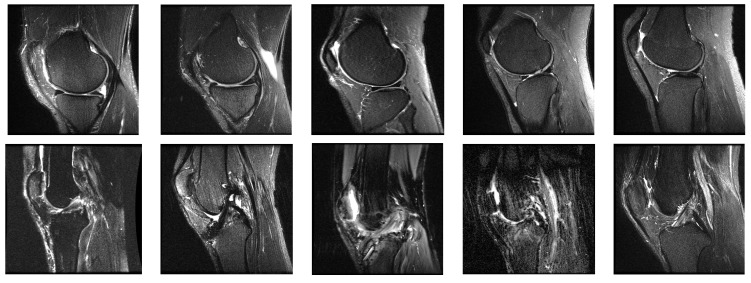
Sample images from the MRNet database.

The proposed system was implemented using Python-OpenCV programming on a personal computer having a core i3 processor with a speed of 2.64 GHz, 4 GB RAM, and a Windows environment. The performance of the proposed CPDCNN was evaluated on the MRNet knee joint MRI dataset [20]. The MRNet dataset consists of MRI images in sagittal T2, coronal T1, and axial PD views with multiple frames that consist of ACL and meniscal tears. We selected specific frames from the dataset in the sagittal T2 view, which consists of a complete view of normal and tear ligaments. Each image in the dataset has a dimension of 256 × 256 pixels. The sample images from the dataset are illustrated in Figure 3. We selected 845 normal and 450 abnormal samples to form the dataset. Out of the total database, 70% of the data were used for training, and 30% of the data were used for testing.

### 4.2. Performance Metrics

The outcomes of the proposed scheme were validated using various quantitative and qualitative metrics such as precision, recall, accuracy, and the F1 score. Equations (5)–(8) are used to calculate precision, recall, accuracy, and the F1 score, respectively.

(5) $$Precision=\frac{TP}{TP+FP}$$

(6) $$Recall=\frac{TN}{TN+FN}$$

(7) $$Accuracy\;\left(\%\right)=\frac{TP+TN}{TP+TN+FP+FN}\times 100$$

(8) $$F1-Score=\frac{2*Precision*Recall}{Precision+Recall}$$

### 4.3. Network Parameters

The parameter specifications and feature maps of the different layers of the proposed CPDCNN knee tear detection system are given in Table 1. The proposed CPDCNN provided 393,216 trainable parameters per parallel arm and a total of 786,434 trainable parameters for knee tear detection. 

**Figure 4:**
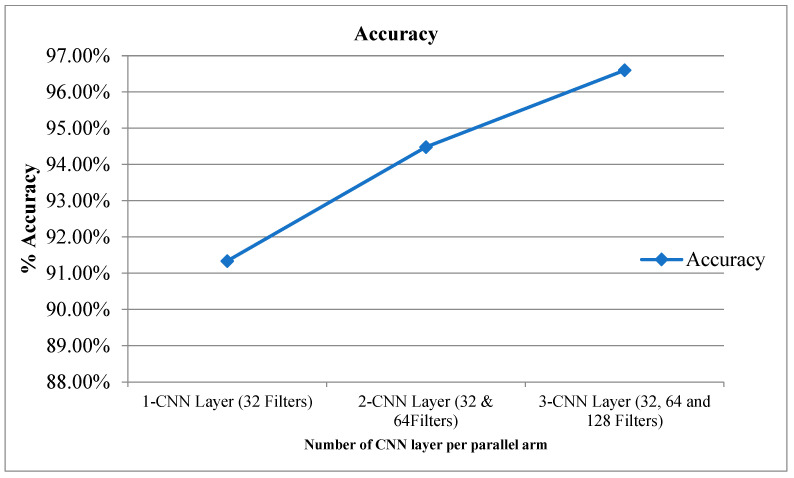
Accuracy for proposed CPDCNN for various layers.

**Figure 5:**
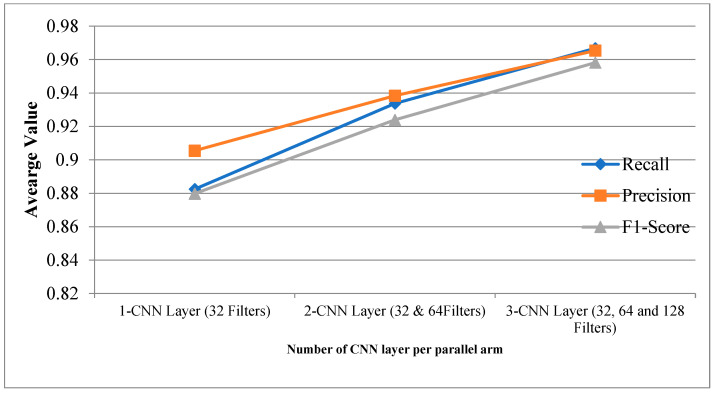
Recall, precision, and F1 score for proposed CPDCNN for various layers.

Table 2 provides the experimental results for the proposed CPDCNN for various CNN layers at each parallel arm. The use of a three-layered CPDCNN provided a better representation of the fine and coarse textures of the knee MRI image, thus helping to characterize such tear injuries from normal knee MRI images. The proposed CDCNN provided accuracy values of 91.34%, 94.48%, and 96.60% for CPDCNNs that included, respectively, one CNN with 32 filters, two CNNs with 32 and 64 filters, and three CNNs with 32, 64, and 128 filters for convolutional operation. Increasing the CNN layers in the parallel arm showed significant improvement in tear detection accuracy because of improvement in deep feature representation. Figure 4 and Figure 5 illustrate the performance of the proposed CPDCNN for different CNNs at each parallel arm.

The outcomes of the proposed CPDCNN-based ACL tear detection were evaluated for the different learning algorithms such as Adam, stochastic gradient descent momentum (SGDM), and root-mean-square propagation (RMSProps) algorithm, as described in Table 3. It had 96.60% accuracy for the 3 × 3 pixels convolutional kernel, which is superior to other windows. The 3 × 3 pixels window provides better spatial connectivity of the local regions of the MRI image that help to characterize the normal and tear textures of the knee MRI image. It provided 96.60%, 95.88%, 94.48%, and 92.92% accuracy values for the 3 × 3 pixels, 5 × 5 pixels, 7 × 7 pixels, and 9 × 9 pixels convolutional filter kernels, respectively. The CPDCNN-Adam provided 96.60% accuracy, which is superior to CPDCNN-SGDM (95.88) and CPDCNN-RMSProps (94.48%). The F1 score represents a good balance between the quantitative (recall) and qualitative (precision) performance of the CPDCNN for knee tear detection. The F1 scores of the proposed CPDCNN were 0.9610, 0.9589, and 0.9415 for Adam, SGDM, and RMSProps learning strategies, respectively.

## 5. Discussion

The performance of the proposed system was compared with previous deep-learning-based ACL tear detection techniques, as shown in Table 3. The proposed system showed significant improvement in ACL tear detection, compared with previous deep-learning-based approaches for ACL tear detection. The proposed CPDCNNN showed an improvement of 1.68%, 3.42%, 6.09%, 7.15%, and 18.86% in ACL tear detection accuracy over DCNN [13], ResNet18 [22], Efficient Net [17], MLP [14], and ResNet50 [21], respectively, for ACL tear detection. The proposed CPDCNN provided a total of 786434 trainable parameters, which are fewer compared with ResNet50 [21], ResNet18 [22], and ResNet [23]. The proposed network’s fewer trainable parameters provide faster training, testing, and implementation feasibility on standalone devices based on microprocessors or microcontrollers.

## 6. Conclusions and Future Scope

Thus, in the proposed system, CPDCNN was presented to improve feature distinctiveness, thus improving the representation capability of the complex knee MRI texture for ACL tear detection using knee MRI images. CPDCNN helps to discriminate between a torn texture and normal textures of the ACL region in knee MRI images and provides better local and global feature representation capability. The proposed CPDCNN had 96.60% accuracy on the Knee MRI dataset, and it helps to minimize the complexity of deep learning frameworks and the problem of hyperparameter tuning. The results of this study revealed that the proposed system had better performance than the previous state-of-the-art deep-learning-based methods for ACL tear detection. In the future, the performance of the proposed approach can be improved by using an augmented dataset. The CPDCNN provided a total of 786,434 trainable parameters, which are lower than the existing state-of-the-art methods, thus helping to minimize the computational complexity of the network. The proposed system yielded 96.60% accuracy for the three-layered CPDCNN for ACL tear detection. This method can be extended to develop a generalized framework for the detection of multiple types and multi-stage knee tear detection using datasets with higher volumes of data.

## Figures and Tables

**Table 1:** Parameter specification of proposed CPDCNN.

Layer	Filter Dimensions	Padding	Stride	Activation Map	Total Trainable Parameters
Input Image				256 × 256 × 1	
Conv11	3 × 3 × 32	[1,1]	[1,1]	256 × 256 × 32	4640
BN11				256 × 256× 32	64
ReLU11				256 × 256 × 32	
MaxPool11			[2,2]	128 × 128 × 32	
Conv12	3 × 3 × 64	[1,1]	[1,1]	128 × 128 × 64	18,496
BN12				128 × 128 × 64	128
ReLU12				128 × 128 × 64	
MaxPool12			[2,2]	64 × 64 × 64	
Conv13	3 × 3 × 128	[1,1]	[1,1]	64 × 64 × 128	73,856
BN13				64 × 64 × 128	256
ReLU13				64 × 64 × 128	
MaxPool13				32 × 32 × 128	
Conv21	3 × 3 × 32	[1,1]	[1,1]	256×256 × 32	4640
BN21				256 × 256 × 32	64
ReLU21				256 × 256 × 32	
MaxPool21			[2,2]	128 × 128 × 32	
Conv22	3 × 3 × 64	[1,1]	[1,1]	128 × 128 × 64	18,496
BN22				128 × 128 × 64	128
ReLU22				128 × 128 × 64	
MaxPool22			[2,2]	64 × 64 × 64	
Conv23	3 × 3 × 128	[1,1]	[1,1]	64 × 64 × 128	73,856
BN23				64 × 64 × 128	256
ReLU23				64 × 64 × 128	
MaxPool23				32 × 32× 128	
Conv31	3 × 3 × 32	[1,1]	[1,1]	256 × 256 × 32	4640
BN31				256 × 256 × 32	64
ReLU31				256 × 256 × 32	
MaxPool31			[2,2]	128 × 128 × 32	
Conv32	3 × 3 × 64	[1,1]	[1,1]	128 × 128 × 64	18,496
BN32				128 × 128 × 64	128
ReLU32				128 × 128 × 64	
MaxPool32			[2,2]	64 × 64 × 64	
Conv33	3 × 3 × 128	[1,1]	[1,1]	64 × 64 × 128	73,856
BN33				64 × 64 × 128	256
ReLU33				64 × 64 × 128	
MaxPool33				32 × 32 × 128	
FC (2 layers)				1 × 1 × 2	786,434
Softmax				1 × 1 × 2	

**Table 2:** Performance of the proposed CPDCNN for different CNN layers.

Layers	Accuracy	Recall	Precision	F1 Score
CPDCNN-Adam	96.60 %	0.9668	0.9554	0.9610
CPDCNN-SGDM	95.88%	0.9589	0.9589	0.9589
CPDCNN-RMSProps	94.48%	0.9448	0.9384	0.9415

**Table 3:** Performance comparison of proposed system with previous ACL tear detection approaches.

Author (Year)	Method	% Accuracy	Total Trainable Parameters
Lai et al. (2018) [14]	MLP	90.15%	
Bien et al. (2018) [13]	DCNN	95.00%	
Tsai et al. (2020) [17]	EfficientNet	91.05%	
Kara et al. (2021) [21]	ResNet50	81.27% (Sagittal view)	39,636,608
Azcona et al. (2020) [22]	ResNet18	93.40%	~11 M
Irmakci et al. (2019) [23]	ResNet, AlexNet and GoogleNet	AUC = 0.956 (ResNet), 0.938 (AlexNet) and 0.890 (googleNet)	ResNet~11 M
Proposed CPDCNN		96.60	786,434

## Data Availability

The dataset used for the work can be found at https://stanfordmlgroup.github.io/competitions/mrnet/ accessed on 1 January 2021.
